# Pediatric hemolysis in emergency departments: Prevalence, risk factors, and clinical implications

**DOI:** 10.1371/journal.pone.0299692

**Published:** 2024-03-21

**Authors:** Nicholas Mielke, Ray Lee, Amit Bahl

**Affiliations:** 1 Creighton University School of Medicine, Omaha, Nebraska, United States of America; 2 Corewell Health Research Institute, Royal Oak, Michigan, United States of America; 3 Corewell Health William Beaumont University Hospital, Royal Oak, Michigan, United States of America; University of Nigeria Faculty of Medical Sciences and Dentistry: University of Nigeria Faculty of Medical Sciences, NIGERIA

## Abstract

**Objective:**

This study aimed to analyze the prevalence, risk factors, and clinical implications of hemolyzed laboratory samples in the pediatric emergency department (ED), a subject on which existing data remains scarce.

**Methods:**

We conducted a multi-site observational cohort analysis of pediatric ED encounters in Metro Detroit, Michigan, United States. The study included participants below 18 years of age who had undergone peripheral intravenous catheter (PIVC) placement and laboratory testing. The primary outcome was the presence of hemolysis, and secondary outcomes included identifying risk factors for hemolysis and assessing the impact of hemolysis on PIVC failure.

**Results:**

Between January 2021 and May 2022, 10,462 ED encounters met inclusion criteria, of which 14.0% showed laboratory evidence of hemolysis. The highest proportion of hemolysis occurred in the infant (age 0–1) population (20.1%). Multivariable regression analysis indicated higher odds of hemolysis for PIVCs placed in the hand/wrist in the toddler (age 2–5) and child (age 6–11) subgroups. PIVCs placed in the hand/wrist also demonstrated higher odds of failure in infants.

**Conclusions:**

Hemolysis in the pediatric ED population is a frequent complication that occurs at similar rates as in adults. PIVCs placed in the hand/wrist were associated with higher odds of hemolysis compared to those placed in the antecubital fossa. Clinicians should consider alternative locations for PIVC placement if clinically appropriate. Further research is needed to better understand the clinical implications of pediatric hemolysis.

## Introduction

Hemolysis, the rupture of red blood cells leading to the release of their intracellular contents, is a well-recognized phenomenon in laboratory medicine [1, 2]. Within the emergency department (ED), where rapid and accurate laboratory results are critical for timely patient management and interventions, hemolysis poses significant challenges. Hemolyzed samples can introduce inaccuracies in laboratory results, increase turnaround time, and potentially lead to delayed or inappropriate clinical decisions [3–5]. Studies indicate that the prevalence of hemolyzed samples in the ED is >15% for most adults, varying based on the population and laboratory tests under consideration [6–9]. Several factors can instigate hemolysis, including improper phlebotomy techniques, extended tourniquet application, vigorous mixing, and unsatisfactory specimen handling [10, 11]. These factors are especially pertinent to ED practice, where blood is often sampled directly from the peripheral intravenous catheter (PIVC), an established risk factor for hemolysis [12–15]. In busy EDs, it is a common practice to draw blood directly from freshly inserted PIVCs for operational reasons as well as to reduce the number of overall needlesticks. As this approach is unlikely to change, other strategies to reduce ED hemolysis need exploration.

While several studies have investigated hemolysis, most of the evidence is from adult ED patients and the current prevention recommendations are based upon moderate strength evidence at best [2]. Additionally, pediatric-specific data on hemolysis remain scarce, leaving a significant gap in the understanding of the prevalence, risk factors, and clinical implications of hemolysis in pediatric laboratory samples [16, 17]. In pediatric ED visits, where obtaining bloodwork is often routine, venous access is a fundamental procedure [18–20]. Blood sampling and needle procedures are often among the most distressing experiences for pediatric patients, a perception partly attributable to the potential for repeated blood draws. These repeated interventions are recognized as particularly traumatic for both the patients and their caregivers [21–24]. Thus, mitigating hemolysis through targeted interventions could significantly improve care for this vulnerable population.

Identifying the specific factors contributing to hemolysis in pediatric laboratory samples is crucial for the development of interventions to enhance patient care. This manuscript seeks to bridge this knowledge gap, examining the prevalence of hemolyzed samples in the pediatric ED setting, identifying associated risk factors, and discussing potential strategies to reduce hemolysis occurrences. Furthermore, we will assess the impact of hemolyzed samples on patient outcomes within this setting.

## Methods

### Study design, setting, and participants

This multi-site observational cohort analysis examined pediatric ED encounters at three EDs in Metro Detroit, Michigan, United States, including one large academic tertiary care center and two suburban EDs. Approximately 300 clinicians were credentialed for pediatric vascular access. Subjects were included if they were below 18 years of age at the time of presentation, had documentation of a PIVC placed while in the ED, and underwent laboratory testing. Exclusion criteria consisted of patients with more than one PIVC, PIVC placement occurred with ultrasound guidance, or if the PIVC was not documented. Participants were split into four groups based on their age at the time of ED presentation. Infants were 0–1 years old, toddlers were 2–5 years old, children were 6–11 years old, and teenagers were 12–17 years old. The local Institutional Review Board approved this study.

### Study definitions

Laboratory tests queried for hemolysis included basic/comprehensive metabolic panel and whole-blood potassium. Only the first set of blood samples drawn through the PIVC were included. Any laboratory-detectable level of hemolysis in any single lab sample was characterized as hemolyzed. Subjects could only have one hemolysis event per PIVC. A history of coagulation disorder was defined as having a past medical history with an International Classification of Diseases, Tenth Revision (ICD-10) code of D65-68.

### Data sources

Collection of data variables occurred through a query of the electronic medical record (EPIC, Verona, Wisconsin) on November 6^th^, 2022. This included demographics (age, sex, race), comorbidities, details pertaining to the ED visit (length of stay), PIVC characteristics (gauge, laterality, location, dwell time, removal reason), and laboratory results (basic metabolic panel, comprehensive metabolic panel, and whole-blood potassium). Throughout the data collection process and thereafter, the authors maintained strict adherence to privacy guidelines, ensuring they did not have access to any information that could potentially identify individual participants.

### Outcomes and measurements

The primary outcome was the presence of hemolysis. Secondary outcomes included identifying risk factors for hemolysis and characterizing the correlation of hemolysis to PIVC failure.

### Statistical analysis

Cases with missing covariates were removed from the analysis. Means and standard deviations were summarized for continuous variables and then compared with student’s t-test/Kruskal-Wallis test. Frequency and percentages were summarized for categorical variables and compared with chi-squared test/Fisher’s exact test. In the primary analysis, we conducted analysis on four stratified pediatric subgroups: infants (0–1), toddlers (2–5), teens (6–11) and teenagers (12–18). Logistic regression models were used for each subgroup to test the association between Hemolyzed outcome (Yes/No) and PIVC insertion location, adjusting for PIVC insertion orientation, sex, and race in the model. Odds ratios (ORs), 95% confidence interval and p-values were reported. In the secondary analysis, only admitted patients were selected. Logistic regression model was used for testing the association between Hemolyzed status and PIVC failure outcome. Unadjusted univariate and multivariate analysis were conducted with results presented. Statistical Analysis was performed on R 4.2.2. Statistical significance was defined as p-value < 0.05.

## Results

Between January 8^th^, 2021, and May 9^th^, 2022, a total of 10,462 encounters met inclusion criteria. 1,461 (14.0%) subjects had laboratory evidence of hemolysis, while 9,001 (86.0%) did not. The majority of the cohort was comprised of ages 12–18 (5,222; 49.9%), followed by ages 6–11 (1,878; 18.0%), age 0–1 (1,710; 16.3%), and ages 2–5 (1,652; 15.8%). Female sex comprised 55.3% of the cohort, which was predominantly White (63.6%). The average length of stay was 62.71 hours and 29.4% of all encounters resulted in hospitalization. The most common PIVC sizes were 20 gauge (45.5%) and 22 gauge (42.9%). 34.2% of all PIVCs failed, which was higher amongst the hemolysis cohort (41.3%) compared to the non-hemolysis cohort (32.9%; p<0.001) (Table 1).

**Table 1:** Demographics and PIVC characteristics of all pediatric patients based on hemolysis.

Variables*	All	Hemolysis		
		Yes	No	
n	10462	1461 (14.0%)	9001 (86.0%)	*p* value
Demographics				
Age, category				<0.001^1^
0–1	1710 (16.3%)	343 (23.5%)	1367 (15.2%)	
2–5	1652 (15.8%)	289 (19.8%)	1363 (15.1%)	
6–11	1878 (18.0%)	210 (14.4%)	1668 (18.5%)	
12–18	5222 (49.9%)	619 (42.4%)	4603 (51.1%)	
Age, years				<0.001^2^
Mean	9.93 (6.43)	8.62 (6.73)	10.14 (6.35)	
Median	11.00 (3.00, 16.00)	8.00 (2.00, 16.00)	12.00 (4.00, 16.00)	
Sex				0.203^3^
Female	5782 (55.3%)	785 (53.7%)	4997 (55.5%)	
Male	4680 (44.7%)	676 (46.3%)	4004 (44.5%)	
Race				0.004^3^
Black or African American	2597 (24.8%)	425 (29.1%)	2172 (24.1%)	
White or Caucasian	6652 (63.6%)	871 (59.6%)	5781 (64.2%)	
Other	1213 (11.6%)	165 (11.3%)	1048 (11.6%)	
ED Disposition				<0.001^3^
Discharge	7387 (70.6%)	905 (61.9%)	6482 (72.0%)	
Admission	3075 (29.4%)	556 (38.1%)	2519 (28.0%)	
Length of stay, hours				0.033^2^
Mean	62.71 (81.25)	57.01 (66.94)	63.98 (84.07)	
Median	43.03 (25.31, 69.47)	40.28 (24.52, 63.95)	43.36 (25.53, 70.26)	
Not available	7262	877	6385	
History of coagulation disorder				0.187^3^
Yes	43 (0.4%)	9 (0.6%)	34 (0.4%)	
No	10419 (99.6%)	1452 (99.4%)	8967 (99.6%)	
Home anticoagulant medication				0.202^3^
Yes	10 (0.1%)	0 (0.0%)	10 (0.1%)	
No	10452 (99.9%)	1461 (100.0%)	8991 (99.9%)	
PIVC Characteristics				
Gauge				<0.001^1^
18	267 (2.6%)	49 (3.4%)	218 (2.4%)	
20	4762 (45.5%)	521 (35.7%)	4241 (47.1%)	
22	4490 (42.9%)	744 (50.9%)	3746 (41.6%)	
24	943 (9.0%)	147 (10.1%)	796 (8.8%)	
Orientation				0.932^3^
Left	3992 (38.2%)	556 (38.1%)	3436 (38.2%)	
Right	6470 (61.8%)	905 (61.9%)	5565 (61.8%)	
Location				<0.001^3^
Antecubital	7371 (71.1%)	845 (58.4%)	6526 (73.2%)	
Forearm	1045 (10.1%)	173 (12.0%)	872 (9.8%)	
Upper Arm	193 (1.9%)	27 (1.9%)	166 (1.9%)	
Hand/Wrist	1515 (14.6%)	355 (24.5%)	1160 (13.0%)	
Lower Leg	25 (0.2%)	2 (0.1%)	23 (0.3%)	
Foot	174 (1.7%)	38 (2.6%)	136 (1.5%)	
Scalp	34 (0.3%)	5 (0.3%)	29 (0.3%)	
Other	5 (0.0%)	2 (0.1%)	3 (0.0%)	
Not documented	100	14	86	
Removal Reason				<0.001^3^
Failure	1716 (34.2%)	316 (41.3%)	1400 (32.9%)	
Therapy Completion	3305 (65.8%)	450 (58.7%)	2855 (67.1%)	
Not documented	5441	695	4746	
Removal Reason Subcategory				<0.001^3^
Therapy Completion	8746 (83.6%)	1145 (78.4%)	7601 (84.4%)	
Dislodgement	129 (1.2%)	25 (1.7%)	104 (1.2%)	
Infection	1 (0.0%)	0 (0.0%)	1 (0.0%)	
Infiltration	145 (1.4%)	29 (2.0%)	116 (1.3%)	
Leaking	147 (1.4%)	27 (1.8%)	120 (1.3%)	
Occlusion	123 (1.2%)	23 (1.6%)	100 (1.1%)	
Phlebitis	10 (0.1%)	1 (0.1%)	9 (0.1%)	
Unclear etiology	1161 (11.1%)	211 (14.4%)	950 (10.6%)	
Dwell Time				<0.001^2^
Mean	18.87 (28.55)	20.35 (25.09)	18.63 (29.07)	
Median	6.08 (3.20, 25.03)	9.18 (3.65, 28.04)	5.82 (3.15, 24.35)	
Not documented	18	3	15	

*For continuous variables, medians (interquartile ranges, IQRs) and means (standard deviation, SD) were presented. For categorical variables, frequencies (percentage) were presented.

^1^Kruskal-Wallis rank sum test

^2^Student’s t-test

^3^Pearson’s Chi-squared test

In the infant (age 0–1) population, the frequency of hemolysis was 20.1%, with the 24-gauge PIVC being the most common (50.4%) (S1 Table). The frequency of hemolysis was lower among the toddler (2–5) population (17.5%), with the most common PIVC being 22-gauge (93.8%) (S2 Table). Similarly, the 22-gauge (76.9%) was the most common PIVC in children (ages 6–11), with 11.2% having hemolysis (S3 Table). Amongst teenagers, the 20-gauge PIVC was the most common (82.3%), with 11.9% of subjects having hemolysis (S4 Table).

The adjusted multivariable regression analysis, in the toddler subgroup, PIVCs placed in the hand/wrist were associated with higher odds of hemolysis (adjusted odds ratio [aOR] 2.51; 95% confidence interval [CI] 1.88–3.34; p<0.001), as well as the lower extremity (aOR 3.77; 95% CI 1.48–8.99; p = 0.0035). Children had higher odds of hemolysis for PIVCs placed in the hand/wrist (aOR 3.08; 95% CI 2.04–4.58; p<0.001) and forearm (aOR 2.00; 95% CI 1.27–3.06; p = 0.0020) compared to the antecubital fossa. In teenagers, male sex compared to female sex (aOR 1.25; 95% CI 1.04–1.49; p = 0.0149) and Black race compared to White race (aOR 1.40; 95% CI 1.16–1.70; p<0.001) had higher odds of hemolysis. Additionally, compared to PIVCs placed in the antecubital fossa, teenagers had higher odds of hemolysis with PIVCs in the forearm (aOR 1.65; 95% CI 1.31–2.08; p<0.001) and hand/wrist (aOR 2.54; 95% CI 1.92–3.34; p<0.001) (Table 2).

**Table 2:** Multivariate analysis for the odds of hemolysis by age group.

Terms	Infants		Toddlers		Children		Teen	
	aOR* (95% CI)	*p* value	aOR* (95% CI)	*p* value	aOR^†^ (95% CI)	*p* value	aOR^†^ (95% CI)	*p* value
Sex								
Female	Reference		Reference		Reference		Reference	
Male	0.9 (0.71–1.15)	0.4073	0.83 (0.64–1.08)	0.1623	0.79 (0.58–1.06)	0.1161	1.25 (1.04–1.49)	0.0149
Race								
White	Reference		Reference		Reference		Reference	
Black	1.27 (0.95–1.67)	0.1014	1.28 (0.94–1.27)	0.1169	1.33 (0.95–1.85)	0.0913	1.4 (1.16–1.7)	<0.001
Other	0.82 (0.56–1.18)	0.3009	1.24 (0.83–1.83)	0.2774	0.91 (0.54–1.47)	0.7155	0.99 (0.73–1.31)	0.9271
Gauge								
18	-	-	-	-	2.91 (0.39–15.75)	0.2307	1.72 (1.22–2.39)	0.0015
20	-	-	-	-	0.85 (0.58–1.23)	0.4033	Reference	
22	-	-	-	-	Reference		1.38 (1.08–1.75)	0.0082
24	-	-	-	-	0.82 (0.12–3.16)	0.8041	2.09 (0.1–16.86)	0.5300
Orientation								
Left	Reference		Reference		Reference		Reference	
Right	0.95 (0.74–1.21)	0.6804	0.97 (0.74–1.27)	0.8329	0.83 (0.61–1.12)	0.2165	1.1 (0.92–1.32)	0.2833
Location								
Antecubital	Reference		Reference		Reference		Reference	
Forearm	0.80 (0.37–1.55)	0.5264	1.50 (0.90–2.40)	0.1072	2.00 (1.27–3.06)	0.0020	1.65 (1.31–2.08)	<0.001
Upper Arm	-	-	0.82 (0.19–2.42)	0.7537	1.00 (0.34–2.35)	0.9971	1.51 (0.84–2.55)	0.1482
Hand/Wrist	1.01 (0.78–1.31)	0.9255	2.51 (1.88–3.34)	<0.001	3.08 (2.04–4.58)	<0.001	2.54 (1.92–3.34)	<0.001
Foot	-	-	-	-	5.03 (0.23–53.3)	0.1902	3.77 (0.17–40.46)	0.2842
Lower extremity	0.85 (0.54–1.29)	0.4495	3.77 (1.48–8.99)	0.0035	-	-	-	-
Scalp	0.73 (0.24–1.78)	0.5289	-	-	-	-	-	-
Other	0.95 (0.21–3.04)	0.9358	-	-	-	-	-	-

Abbreviations: aOR = adjusted odds ratio; CI = confidence interval

*Adjusted multivariable logistic regression analysis for age-specific subgroup, adjusted for sex, race, orientation, and location.

^†^Adjusted multivariable logistic regression analysis for age specific subgroup, adjusted for sex, race, gauge, orientation, and location.

The odds of failure were assessed via an adjusted multivariate analysis. This did not reveal the presence of hemolysis to significantly impact the odds of failure amongst any of the four age groups. However, in infants, PIVCs placed in the hand/wrist had higher odds of failure (aOR 1.60; 95% CI 1.11–2.33; p = 0.0126) compared to the antecubital fossa. In children, PIVCs placed in the upper arm had lower odds of failure (aOR 0.34; 95% CI 0.11–0.90; p = 0.0419) compared to the antecubital fossa (Table 3).

**Table 3:** Multivariate analysis for the odds of failure by age group.

Terms	Infants		Toddlers		Children		Teen	
	aOR* (95% CI)	p value	aOR* (95% CI)	p value	aOR* (95% CI)	p value	aOR* (95% CI)	p value
Sex								
Female	Reference		Reference		Reference		Reference	
Male	0.76 (0.56–1.03)	0.0785	0.88 (0.61–1.25)	0.4704	1 (0.68–1.48)	0.9905	0.8 (0.59–1.09)	0.154
Race								
White	Reference		Reference		Reference		Reference	
Black	1.17 (0.81–1.68)	0.4086	1.41 (0.95–2.09)	0.0889	1.33 (0.86–2.06)	0.2022	1.37 (0.97–1.94)	0.0698
Other	1.27 (0.82–1.96)	0.2808	0.93 (0.51–1.7)	0.8204	0.87 (0.46–1.64)	0.6777	0.88 (0.53–1.44)	0.6112
Gauge								
18	-	-	-	-	1.58 (0.17–15.2)	0.6728	2.2 (1.19–4.15)	0.0133
20	2.29 (0.21–50.81)	0.5064	1.2 (0.45–3.2)	0.7061	0.8 (0.49–1.28)	0.3578	Reference	
22	1.24 (0.88–1.76)	0.2165	Reference		Reference		1.34 (0.87–2.06)	0.1794
24	Reference		1.78 (0.71–4.7)	0.2227	1.19 (0.13–11.52)	0.8725	-	-
Orientation								
Left	Reference		Reference		Reference		Reference	
Right	1.23 (0.89–1.69)	0.2096	1.07 (0.74–1.56)	0.7179	1.25 (0.84–1.86)	0.2781	1.11 (0.8–1.55)	0.5149
Location								
Antecubital	Reference		Reference		Reference		Reference	
Forearm	1.48 (0.65–3.44)	0.3518	1.31 (0.67–2.58)	0.4263	1.08 (0.59–1.97)	0.8031	0.84 (0.56–1.27)	0.4169
Upper Arm	2.15 (0.55–10.44)	0.2877	3.54 (1–16.47)	0.0662	0.34 (0.11–0.9)	0.0419	1.62 (0.77–3.49)	0.2052
Hand/Wrist	1.6 (1.11–2.33)	0.0126	1.2 (0.78–1.83)	0.4126	1.24 (0.72–2.13)	0.435	1.53 (0.99–2.37)	0.0574
Lower Leg	0.95 (0.34–2.59)	0.9168	-	-	-	-	-	-
Foot	-	-	3.65 (1–17.22)	0.0643	1.1 (0.04–28.49)	0.9469	-	-
Scalp	1.73 (0.79–3.87)	0.1761	-	-	-	-	-	-
Hemolysis								
No	Reference		Reference		Reference		Reference	
Yes	1.19 (0.81–1.75)	0.3874	1.35 (0.88–2.08)	0.1668	1.28 (0.74–2.2)	0.3711	1.13 (0.76–1.66)	0.5491

Abbreviations: aOR = adjusted odds ratio; CI = confidence interval

*Adjusted multivariable logistic regression analysis for age-specific subgroup, adjusted for sex, race, gauge, orientation, location, and hemolysis

## Discussion

This study of over 10,000 pediatric patients across a nearly 18-month period revealed an overall hemolysis rate of 14.0%, with the highest proportion occurring in the infant population at 20.1%. This study offers an up-to-date look at hemolysis rates in a large pediatric population. Thus far in pediatrics, the evidence on this topic has been scant, with only two small older studies describing the problem. Bush et al. published a prospective study in 2010 of 221 pediatric patients and found a hemolysis rate of 13%, and in the same year, Berger-Achituv et al. published a prospective study on 40 pediatric patients and identified only one case of hemolysis [16, 17]. Therefore, given the extremely limited prior evidence, in essence this study establishes the baseline for hemolysis in pediatric laboratory samples. Further, unlike the adult population in which similar caliber venous anatomy allows for pooling and analysis as a solitary group, stark venous differences and anatomical considerations based on age must be considered in the pediatric population. Thus, alongside a large sample size, an important strength of this study over prior works is the segmenting of the population into four cohorts to allow for a more robust analysis accounting for the unique anatomical variations among the age groups. Consistent with previous pediatric research, the literature supports such stratification as conventional and appropriate [25–28].

As hemolysis is a frequent occurrence, this could result in care delays and possibly necessitate additional blood draws [29]. As blood sampling in pediatrics is already a difficult and complex procedure, potentially adding needlesticks for additional draws only heightens patient anxiety and discomfort with the ED encounter [30]. Therefore, it is important for clinicians to take steps to reduce this complication. This study identifies risk factors for hemolysis and actionable strategies for prevention. One important consideration is site selection. PIVC’s inserted in the hand or wrist were found to significantly increase the likelihood of hemolysis in pediatric laboratory samples among patients who are at least a year old, mirroring findings from a recent study by the authors in adult populations [9]. Moreover, the correlation between hand/wrist PIVC placements and hemolysis underscores the Infusion Nursing Society’s guidelines to avoid such placements due to the heightened risk of premature failure [31]. Despite the infant population not demonstrating increased odds of hemolysis for hand/wrist PIVCs, those PIVC’s did demonstrate increased odds of failure. Therefore, our results suggest that hand/wrist PIVC placement should not be the primary option for any pediatric age group, especially those facing hospital admission. However, as options for viable insertion sites are often limited in pediatrics, particularly among infants and toddlers, escalation to advanced techniques like illumination or ultrasound guidance may enhance venous visibility in these circumstances [32].

Another important risk factor for hemolysis discovered in this study was that catheter gauge (i.e., catheter diameter) significantly affected the odds of hemolysis in the adolescent population. Higher hemolysis rates were seen with 18-gauge and 22-gauge catheters compared to 20-gauge. This finding is congruent with prior studies on adults, but as most of these investigations were smaller-scale studies and published over two decades ago, the Emergency Nurses Association has not made any firm recommendations regarding catheter diameter and hemolysis in their Clinical Practice Guidelines [2]. Nevertheless, this newer and larger dataset should help substantiate this finding and help shape future clinical guidelines. Interestingly, in contrast to adult studies, hemolysis was not linked with increased PIVC failure rates [9]. A plausible reason might be that pediatric PIVCs typically have shorter dwell times than those in adults. Furthermore, PIVC placement decisions in pediatrics are often made with extra deliberation due to potential adverse effects on both the patient and the caregiver [21–23]. Given the important clinical implications of hemolysis triggering PIVC failure, further research on this topic is warranted in the pediatric population.

## Limitations

While the investigation yields insightful findings, it is important to acknowledge certain limitations that potentially impact the results and interpretations. First, the research is subject to potential selection bias due to the non-random selection of participants. The use of convenience sampling could limit the generalizability of the findings to the broader pediatric population. However, a very large sample size likely mitigates this limitation. Second, the retrospective study design inherently limits the capacity to control for unobserved confounding variables. Consequently, the observed associations should not be construed as cause-and-effect relationships. Third, the study adopted a stratification of the pediatric population into four cohorts based on age-related venous and anatomical differences. While beneficial for a nuanced and clinically relevant analysis, this approach restricts the sample size in each cohort, which may not be powered to detect effects when they may exist. Lastly, this study did not assess the precise impact on delays in care, rate of redraw, or medical errors due to hemolysis. This aspect underscores the need for further research to fully understand the implications of our findings in the context of real-world pediatric clinical practice.

## Conclusions

Hemolysis in the pediatric population appears to occur frequently and at a similar rate to the adult population. Compared to PIVCs placed in the antecubital fossa, PIVCs in the hand/wrist demonstrate higher odds of hemolysis, and clinicians should consider placing PIVCs in alternative locations if clinically appropriate. Further studies are needed to better understand the clinical implications of pediatric hemolysis.

## Supporting information

S1 Table Demographics and PIVC characteristics of infants (age 0–1) based on hemolysis. (DOCX)

S2 Table Demographics and PIVC characteristics of toddlers (age 2–5) based on hemolysis. (DOCX)

S3 Table Demographics and PIVC characteristics of children (age 6–11) based on hemolysis. (DOCX)

S4 Table Demographics and PIVC characteristics of teenagers (age 12–17) based on hemolysis. (DOCX)
