# Supplementary material for: Pediatric hemolysis in emergency departments: Prevalence, risk factors, and clinical implications
Source: PLoS One. 2024 Mar 21;19(3):e0299692. doi: 10.1371/journal.pone.0299692 (PMC10956767; doi:10.1371/journal.pone.0299692)
Supplement: S1 Table — (DOCX) [file pone.0299692.s001.docx]

Supplementary Table 1. Demographics and PIVC characteristics of infants (age 0-1) based on hemolysis.

|  |  |  |  | Hemolysis | |  |
| --- | --- | --- | --- | --- | --- | --- |
| Variables* | | | All | Yes | No | *p* value |
|  |  | n | 1710 | 343 (20.1%) | 1367 (79.9%) |  |
| Demographics | | |  |  |  |  |
|  | Age, years | |  |  |  | 0.004^1^ |
|  |  | Mean | 0.41 (0.49) | 0.48 (0.50) | 0.40 (0.49) |  |
|  | Sex | |  |  |  | 0.323^2^ |
|  |  | Female | 772 (45.1%) | 163 (47.5%) | 609 (44.6%) |  |
|  |  | Male | 938 (54.9%) | 180 (52.5%) | 758 (55.4%) |  |
|  | Race | |  |  |  | 0.719^2^ |
|  |  | Black or African American | 387 (22.6%) | 91 (26.5%) | 296 (21.7%) |  |
|  |  | White or Caucasian | 1079 (63.1%) | 210 (61.2%) | 869 (63.6%) |  |
|  |  | Other | 244 (14.3%) | 42 (12.2%) | 202 (14.8%) |  |
|  | ED Disposition | |  |  |  | 0.402^2^ |
|  |  | Discharge | 803 (47.0%) | 168 (49.0%) | 635 (46.5%) |  |
|  |  | Admission | 907 (53.0%) | 175 (51.0%) | 732 (53.5%) |  |
|  | Length of stay, hours | |  |  |  | 0.048^1^ |
|  |  | Mean | 52.69 (57.03) | 47.68 (43.44) | 53.94 (59.89) |  |
|  |  | Median | 40.60 (24.13, 58.60) | 32.84 (22.63, 54.11) | 41.45 (24.55, 60.02) |  |
|  |  | Not available | 765 | 155 | 610 |  |
| PIVC Characteristics | | |  |  |  |  |
|  | Gauge | |  |  |  | <0.001^3^ |
|  |  | 18 | 0 (0.0%) | 0 (0.0%) | 0 (0.0%) |  |
|  |  | 20 | 14 (0.8%) | 3 (0.9%) | 11 (0.8%) |  |
|  |  | 22 | 834 (48.8%) | 205 (59.8%) | 629 (46.0%) |  |
|  |  | 24 | 862 (50.4%) | 135 (39.4%) | 727 (53.2%) |  |
|  | Orientation | |  |  |  | 0.602^2^ |
|  |  | Left | 662 (38.7%) | 137 (39.9%) | 525 (38.4%) |  |
|  |  | Right | 1048 (61.3%) | 206 (60.1%) | 842 (61.6%) |  |
|  | Location | |  |  |  | 0.633^2^ |
|  |  | Antecubital | 789 (46.5%) | 162 (47.6%) | 627 (46.3%) |  |
|  |  | Forearm | 58 (3.4%) | 10 (2.9%) | 48 (3.5%) |  |
|  |  | Upper Arm | 14 (0.8%) | 3 (0.9%) | 11 (0.8%) |  |
|  |  | Hand/Wrist | 631 (37.2%) | 130 (38.2%) | 501 (37.0%) |  |
|  |  | Lower Leg | 22 (1.3%) | 2 (0.6%) | 20 (1.5%) |  |
|  |  | Foot | 147 (8.7%) | 28 (8.2%) | 119 (8.8%) |  |
|  |  | Scalp | 33 (1.9%) | 5 (1.5%) | 28 (2.1%) |  |
|  |  | Other | 1 (0.1%) | 0 (0.0%) | 1 (0.1%) |  |
|  |  | Not documented | 15 | 3 | 12 |  |
|  | Removal Reason | |  |  |  | 0.672^2^ |
|  |  | Failure | 476 (45.8%) | 97 (47.1%) | 379 (45.4%) |  |
|  |  | Therapy Completion | 564 (54.2%) | 109 (52.9%) | 455 (54.6%) |  |
|  |  | Not documented | 670 | 137 | 533 |  |
|  | Removal Reason Subcategory | | |  |  | 0.520^2^ |
|  |  | Therapy Completion | 1234 (72.2%) | 246 (71.7%) | 988 (72.3%) |  |
|  |  | Dislodgement | 59 (3.5%) | 8 (2.3%) | 51 (3.7%) |  |
|  |  | Infection | 0 (0.0%) | 0 (0.0%) | 0 (0.0%) |  |
|  |  | Infiltration | 60 (3.5%) | 7 (2.0%) | 53 (3.9%) |  |
|  |  | Leaking | 55 (3.2%) | 11 (3.2%) | 44 (3.2%) |  |
|  |  | Occlusion | 55 (3.2%) | 11 (3.2%) | 44 (3.2%) |  |
|  |  | Phlebitis | 3 (0.2%) | 0 (0.0%) | 3 (0.2%) |  |
|  |  | Unclear etiology | 244 (14.3%) | 60 (17.5%) | 184 (13.5%) |  |
|  | Dwell Time | |  |  |  | 0.945^1^ |
|  |  | Mean | 22.59 (24.27) | 21.89 (23.06) | 22.77 (24.56) |  |
|  |  | Median | 15.82 (3.85, 34.98) | 16.75 (4.01, 32.00) | 15.57 (3.80, 35.20) |  |
|  |  | Not documented | 4 | 1 | 3 |  |

*For continuous variables, medians (interquartile ranges, IQRs) and means (standard deviation, SD) were presented. For categorical variables, frequencies (percentage) were presented.

^1^Student’s t-test

^2^Pearson’s Chi-squared test

^3^Kruskal-Wallis rank sum test
