# Supplementary material for: Pediatric hemolysis in emergency departments: Prevalence, risk factors, and clinical implications
Source: PLoS One. 2024 Mar 21;19(3):e0299692. doi: 10.1371/journal.pone.0299692 (PMC10956767; doi:10.1371/journal.pone.0299692)
Supplement: S2 Table — (DOCX) [file pone.0299692.s002.docx]

Supplementary Table 2. Demographics and PIVC characteristics of toddlers (age 2-5) based on hemolysis.

|  |  |  |  | Hemolysis | |  |
| --- | --- | --- | --- | --- | --- | --- |
| Variables* | | | All | Yes | No | *p* value |
|  |  | n | 1652 | 289 (17.5%) | 1363 (82.5%) |  |
| Demographics | | |  |  |  |  |
|  | Age, years | |  |  |  | 0.034^1^ |
|  |  | Mean | 3.26 (1.13) | 3.14 (1.16) | 3.29 (1.13) |  |
|  | Sex | |  |  |  | 0.077^2^ |
|  |  | Female | 751 (45.5%) | 145 (50.2%) | 606 (44.5%) |  |
|  |  | Male | 901 (54.5%) | 144 (49.8%) | 757 (55.5%) |  |
|  | Race | |  |  |  | 0.137^2^ |
|  |  | Black or African American | 392 (23.7%) | 76 (26.3%) | 316 (23.2%) |  |
|  |  | White or Caucasian | 1059 (64.1%) | 174 (60.2%) | 885 (64.9%) |  |
|  |  | Other | 201 (12.2%) | 39 (13.5%) | 162 (11.9%) |  |
|  | ED Disposition | |  |  |  | 0.002^2^ |
|  |  | Discharge | 1013 (61.3%) | 154 (53.3%) | 859 (63.0%) |  |
|  |  | Admission | 639 (38.7%) | 135 (46.7%) | 504 (37.0%) |  |
|  | Length of stay, hours | |  |  |  | 0.439^1^ |
|  |  | Mean | 47.54 (40.69) | 48.63 (39.75) | 47.25 (40.97) |  |
|  |  | Median | 34.97 (23.65, 53.91) | 37.37 (24.08, 54.90) | 34.01 (23.63, 53.12) |  |
|  |  | Not available | 988 | 149 | 839 |  |
| PIVC Characteristics | | |  |  |  |  |
|  | Gauge | |  |  |  | 0.241^3^ |
|  |  | 18 | 1 (0.1%) | 0 (0.0%) | 1 (0.1%) |  |
|  |  | 20 | 40 (2.4%) | 10 (3.5%) | 30 (2.2%) |  |
|  |  | 22 | 1550 (93.8%) | 270 (93.4%) | 1280 (93.9%) |  |
|  |  | 24 | 61 (3.7%) | 9 (3.1%) | 52 (3.8%) |  |
|  | Orientation | |  |  |  | 0.790^2^ |
|  |  | Left | 606 (36.7%) | 108 (37.4%) | 498 (36.5%) |  |
|  |  | Right | 1046 (63.3%) | 181 (62.6%) | 865 (63.5%) |  |
|  | Location | |  |  |  | <0.001^2^ |
|  |  | Antecubital | 1096 (67.0%) | 148 (51.6%) | 948 (70.3%) |  |
|  |  | Forearm | 118 (7.2%) | 23 (8.0%) | 95 (7.0%) |  |
|  |  | Upper Arm | 25 (1.5%) | 3 (1.0%) | 22 (1.6%) |  |
|  |  | Hand/Wrist | 374 (22.9%) | 105 (36.6%) | 269 (20.0%) |  |
|  |  | Lower Leg | 1 (0.1%) | 0 (0.0%) | 1 (0.1%) |  |
|  |  | Foot | 21 (1.3%) | 8 (2.8%) | 13 (1.0%) |  |
|  |  | Scalp | 0 (0.0%) | 0 (0.0%) | 0 (0.0%) |  |
|  |  | Other | 0 (0.0%) | 0 (0.0%) | 0 (0.0%) |  |
|  |  | Not documented | 17 | 2 | 15 |  |
|  | Removal Reason | |  |  |  | 0.008^2^ |
|  |  | Failure | 317 (35.7%) | 74 (44.6%) | 243 (33.7%) |  |
|  |  | Therapy Completion | 570 (64.3%) | 92 (55.4%) | 478 (66.3%) |  |
|  |  | Not documented | 765 | 123 | 642 |  |
|  | Removal Reason Subcategory | | |  |  | 0.003^2^ |
|  |  | Therapy Completion | 1335 (80.8%) | 215 (74.4%) | 1120 (82.2%) |  |
|  |  | Dislodgement | 28 (1.7%) | 6 (2.1%) | 22 (1.6%) |  |
|  |  | Infection | 0 (0.0%) | 0 (0.0%) | 0 (0.0%) |  |
|  |  | Infiltration | 29 (1.8%) | 8 (2.8%) | 21 (1.5%) |  |
|  |  | Leaking | 19 (1.2%) | 4 (1.4%) | 15 (1.1%) |  |
|  |  | Occlusion | 25 (1.5%) | 7 (2.4%) | 18 (1.3%) |  |
|  |  | Phlebitis | 1 (0.1%) | 0 (0.0%) | 1 (0.1%) |  |
|  |  | Unclear etiology | 215 (13.0%) | 49 (17.0%) | 166 (12.2%) |  |
|  | Dwell Time | |  |  |  | 0.093^1^ |
|  |  | Mean | 19.57 (24.17) | 19.84 (22.00) | 19.52 (24.61) |  |
|  |  | Median | 8.47 (3.47, 26.25) | 15.13 (3.91, 26.79) | 7.47 (3.37, 26.03) |  |
|  |  | Not documented | 3 | 1 | 2 |  |

*For continuous variables, medians (interquartile ranges, IQRs) and means (standard deviation, SD) were presented. For categorical variables, frequencies (percentage) were presented.

^1^Student’s t-test

^2^Pearson’s Chi-squared test

^3^Kruskal-Wallis rank sum test
