# Supplementary material for: Pediatric hemolysis in emergency departments: Prevalence, risk factors, and clinical implications
Source: PLoS One. 2024 Mar 21;19(3):e0299692. doi: 10.1371/journal.pone.0299692 (PMC10956767; doi:10.1371/journal.pone.0299692)
Supplement: S3 Table — (DOCX) [file pone.0299692.s003.docx]

Supplementary Table 3. Demographics and PIVC characteristics of children (age 6-11) based on hemolysis.

|  |  |  |  | Hemolysis | |  |
| --- | --- | --- | --- | --- | --- | --- |
| Variables* | | | All | Yes | No | *p* value |
|  |  | n | 1878 | 210 (11.2%) | 1668 (88.8%) |  |
| Demographics | | |  |  |  |  |
|  | Age, years | |  |  |  | 0.867^1^ |
|  |  | Mean | 8.49 (1.74) | 8.51 (1.75) | 8.48 (1.74) |  |
|  | Sex | |  |  |  | 0.115^2^ |
|  |  | Female | 950 (50.6%) | 117 (55.7%) | 833 (49.9%) |  |
|  |  | Male | 928 (49.4%) | 93 (44.3%) | 835 (50.1%) |  |
|  | Race | |  |  |  | 0.337^2^ |
|  |  | Black or African American | 453 (24.1%) | 61 (29.0%) | 392 (23.5%) |  |
|  |  | White or Caucasian | 1217 (64.8%) | 128 (61.0%) | 1089 (65.3%) |  |
|  |  | Other | 208 (11.1%) | 21 (10.0%) | 187 (11.2%) |  |
|  | ED Disposition | |  |  |  | <0.001^2^ |
|  |  | Discharge | 1325 (70.6%) | 126 (60.0%) | 1199 (71.9%) |  |
|  |  | Admission | 553 (29.4%) | 84 (40.0%) | 469 (28.1%) |  |
|  | Length of stay, hours | |  |  |  | 0.228^1^ |
|  |  | Mean | 63.54 (88.16) | 51.36 (50.84) | 65.69 (93.11) |  |
|  |  | Median | 41.92 (26.25, 68.07) | 36.21 (26.50, 57.48) | 42.25 (26.07, 69.53) |  |
|  |  | Not available | 1307 | 124 | 1183 |  |
| PIVC Characteristics | | |  |  |  |  |
|  | Gauge | |  |  |  | 0.285^3^ |
|  |  | 18 | 6 (0.3%) | 2 (1.0%) | 4 (0.2%) |  |
|  |  | 20 | 412 (21.9%) | 39 (18.6%) | 373 (22.4%) |  |
|  |  | 22 | 1444 (76.9%) | 167 (79.5%) | 1277 (76.6%) |  |
|  |  | 24 | 16 (0.9%) | 2 (1.0%) | 14 (0.8%) |  |
|  | Orientation | |  |  |  | 0.249^2^ |
|  |  | Left | 666 (35.5%) | 82 (39.0%) | 584 (35.0%) |  |
|  |  | Right | 1212 (64.5%) | 128 (61.0%) | 1084 (65.0%) |  |
|  | Location | |  |  |  | <0.001^2^ |
|  |  | Antecubital | 1465 (78.4%) | 133 (63.3%) | 1332 (80.3%) |  |
|  |  | Forearm | 174 (9.3%) | 30 (14.3%) | 144 (8.7%) |  |
|  |  | Upper Arm | 58 (3.1%) | 5 (2.4%) | 53 (3.2%) |  |
|  |  | Hand/Wrist | 167 (8.9%) | 40 (19.0%) | 127 (7.7%) |  |
|  |  | Foot | 3 (0.2%) | 1 (0.5%) | 2 (0.1%) |  |
|  |  | Scalp | 1 (0.1%) | 0 (0.0%) | 1 (0.1%) |  |
|  |  | Other | 1 (0.1%) | 1 (0.5%) | 0 (0.0%) |  |
|  |  | Not documented | 9 | 0 | 9 |  |
|  | Removal Reason | |  |  |  | 0.019^2^ |
|  |  | Failure | 291 (32.1%) | 45 (42.1%) | 246 (30.8%) |  |
|  |  | Therapy Completion | 616 (67.9%) | 62 (57.9%) | 554 (69.2%) |  |
|  |  | Not documented | 971 | 103 | 868 |  |
|  | Removal Reason Subcategory | | |  |  | 0.013^2^ |
|  |  | Therapy Completion | 1587 (84.5%) | 165 (78.6%) | 1422 (85.3%) |  |
|  |  | Dislodgement | 9 (0.5%) | 3 (1.4%) | 6 (0.4%) |  |
|  |  | Infection | 0 (0.0%) | 0 (0.0%) | 0 (0.0%) |  |
|  |  | Infiltration | 19 (1.0%) | 3 (1.4%) | 16 (1.0%) |  |
|  |  | Leaking | 21 (1.1%) | 2 (1.0%) | 19 (1.1%) |  |
|  |  | Occlusion | 17 (0.9%) | 3 (1.4%) | 14 (0.8%) |  |
|  |  | Phlebitis | 2 (0.1%) | 0 (0.0%) | 2 (0.1%) |  |
|  |  | Unclear etiology | 223 (11.9%) | 34 (16.2%) | 189 (11.3%) |  |
|  | Dwell Time | |  |  |  | 0.014^1^ |
|  |  | Mean | 19.40 (29.63) | 19.94 (22.84) | 19.33 (30.38) |  |
|  |  | Median | 5.93 (3.15, 25.680) | 11.05 (3.77, 28.01) | 5.78 (3.08, 25.42) |  |
|  |  | Not documented | 4 | 0 | 4 |  |

*For continuous variables, medians (interquartile ranges, IQRs) and means (standard deviation, SD) were presented. For categorical variables, frequencies (percentage) were presented.

^1^Student’s t-test

^2^Pearson’s Chi-squared test

^3^Kruskal-Wallis rank sum test
