# Supplementary material for: Pediatric hemolysis in emergency departments: Prevalence, risk factors, and clinical implications
Source: PLoS One. 2024 Mar 21;19(3):e0299692. doi: 10.1371/journal.pone.0299692 (PMC10956767; doi:10.1371/journal.pone.0299692)
Supplement: S4 Table — (DOCX) [file pone.0299692.s004.docx]

Supplementary Table 4. Demographics and PIVC characteristics of teenagers (age 12-17) based on hemolysis.

|  |  |  |  | Hemolysis | |  |
| --- | --- | --- | --- | --- | --- | --- |
| Variables* | | | All | Yes | No | *p* value |
|  |  | n | 5222 | 619 (11.9%) | 4603 (88.1%) |  |
| Demographics | | |  |  |  |  |
|  | Age, years | |  |  |  | 0.429^1^ |
|  |  | Mean | 15.67 (1.94) | 15.74 (1.90) | 15.66 (1.94) |  |
|  | Sex | |  |  |  | 0.004^2^ |
|  |  | Female | 3309 (63.4%) | 360 (58.2%) | 2949 (64.1%) |  |
|  |  | Male | 1913 (36.6%) | 259 (41.8%) | 1654 (35.9%) |  |
|  | Race | |  |  |  | 0.018^2^ |
|  |  | Black or African American | 1365 (26.1%) | 197 (31.8%) | 1168 (25.4%) |  |
|  |  | White or Caucasian | 3297 (63.1%) | 359 (58.0%) | 2938 (63.8%) |  |
|  |  | Other | 560 (10.7%) | 63 (10.2%) | 497 (10.8%) |  |
|  | ED Disposition | |  |  |  | <0.001^2^ |
|  |  | Discharge | 4246 (81.3%) | 457 (73.8%) | 3789 (82.3%) |  |
|  |  | Admission | 976 (18.7%) | 162 (26.2%) | 814 (17.7%) |  |
|  | Length of stay, hours | |  |  |  | 0.357^1^ |
|  |  | Mean | 81.40 (108.13) | 77.08 (100.97) | 82.26 (109.54) |  |
|  |  | Median | 49.37 (28.31, 95.15) | 48.56 (28.21, 92.13) | 49.37 (28.36, 95.59) |  |
|  |  | Not available | 4202 | 449 | 3753 |  |
| PIVC Characteristics | | |  |  |  |  |
|  | Gauge | |  |  |  | <0.001^3^ |
|  |  | 18 | 260 (5.0%) | 47 (7.6%) | 213 (4.6%) |  |
|  |  | 20 | 4296 (82.3%) | 469 (75.8%) | 3827 (83.1%) |  |
|  |  | 22 | 662 (12.7%) | 102 (16.5%) | 560 (12.2%) |  |
|  |  | 24 | 4 (0.1%) | 1 (0.2%) | 3 (0.1%) |  |
|  | Orientation | |  |  |  | 0.190^2^ |
|  |  | Left | 2058 (39.4%) | 229 (37.0%) | 1829 (39.7%) |  |
|  |  | Right | 3164 (60.6%) | 390 (63.0%) | 2774 (60.3%) |  |
|  | Location | |  |  |  | <0.001^2^ |
|  |  | Antecubital | 4021 (77.9%) | 402 (65.9%) | 3619 (79.5%) |  |
|  |  | Forearm | 695 (13.5%) | 110 (18.0%) | 585 (12.8%) |  |
|  |  | Upper Arm | 96 (1.9%) | 16 (2.6%) | 80 (1.8%) |  |
|  |  | Hand/Wrist | 343 (6.6%) | 80 (13.1%) | 263 (5.8%) |  |
|  |  | Lower Leg | 2 (0.0%) | 0 (0.0%) | 2 (0.0%) |  |
|  |  | Foot | 3 (0.1%) | 1 (0.2%) | 2 (0.0%) |  |
|  |  | Scalp | 0 (0.0%) | 0 (0.0%) | 0 (0.0%) |  |
|  |  | Other | 3 (0.1%) | 1 (0.2%) | 2 (0.0%) |  |
|  |  | Not documented | 59 | 9 | 50 |  |
|  | Removal Reason | |  |  |  | 0.017^2^ |
|  |  | Failure | 632 (28.9%) | 100 (34.8%) | 532 (28.0%) |  |
|  |  | Therapy Completion | 1555 (71.1%) | 187 (65.2%) | 1368 (72.0%) |  |
|  |  | Not documented | 3035 | 332 | 2703 |  |
|  | Removal Reason Subcategory | | |  |  | 0.002^2^ |
|  |  | Therapy Completion | 4590 (87.9%) | 519 (83.8%) | 4071 (88.4%) |  |
|  |  | Dislodgement | 33 (0.6%) | 8 (1.3%) | 25 (0.5%) |  |
|  |  | Infection | 1 (0.0%) | 0 (0.0%) | 1 (0.0%) |  |
|  |  | Infiltration | 37 (0.7%) | 11 (1.8%) | 26 (0.6%) |  |
|  |  | Leaking | 52 (1.0%) | 10 (1.6%) | 42 (0.9%) |  |
|  |  | Occlusion | 26 (0.5%) | 2 (0.3%) | 24 (0.5%) |  |
|  |  | Phlebitis | 4 (0.1%) | 1 (0.2%) | 3 (0.1%) |  |
|  |  | Unclear etiology | 479 (9.2%) | 68 (11.0%) | 411 (8.9%) |  |
|  | Dwell Time | |  |  |  | <0.001^1^ |
|  |  | Mean | 17.24 (30.55) | 19.87 (28.10) | 16.89 (30.85) |  |
|  |  | Median | 5.15 (3.03, 21.03) | 6.40 (3.13, 26.89) | 5.02 (3.00, 20.20) |  |
|  |  | Not documented | 7 | 1 | 6 |  |

Abbreviations: ED=emergency department; PIVC=peripheral intravenous catheter.

*For continuous variables, medians (interquartile ranges, IQRs) and means (standard deviation, SD) were presented. For categorical variables, frequencies (percentage) were presented.

^1^Student’s t-test

^2^Pearson’s Chi-squared test

^3^Kruskal-Wallis rank sum test
